# Supplementary material for: The optimum parameters and neuroimaging mechanism of repetitive transcranial magnetic stimulation to post-stroke cognitive impairment, a protocol of an orthogonally-designed randomized controlled trial
Source: PLoS One. 2022 Jul 21;17(7):e0271283. doi: 10.1371/journal.pone.0271283 (PMC9302729; doi:10.1371/journal.pone.0271283)
Supplement: S2 File — (PDF) [file pone.0271283.s002.pdf]

四川大学华西医院生物医学伦理审查委员会批件

2020年 审(1121)号

科室（专业：	康复医学科/康复医学系	项目负责人姓名及职称：	李凌鑫/主治医师
项目名称	重复经颅磁刺激治疗卒中后认知障碍参数优化及神经影像学研究		
研究方案	版本号：V2.0	版本日期：2020年11月23日	
知情同意书	版本号：1.0	版本日期：2020年10月10日	
招募广告	无		

审查意见：

1. 研究者资质符合伦理要求。
2. 研究方案及知情同意书基本符合伦理要求。

审查结果：■批准　□修改后批准　□修改后再审　□不批准　□暂停或者终止研究

持续审查频率：　□3 个月/3months　□6 个月/6months　■1 年/1year　□不适用/NA

请遵循我国相关法律、法规和规章（《涉及人的生物医学研究伦理审查办法》等）以及WMA《赫尔辛基宣言》和CIOMS《人体生物医学研究国际道德指南》，遵循伦理审查委员会批准的方案和知情同意书开展临床试验（研究），保护受试者的健康与权利。

请严格执行《中华人民共和国人类遗传资源管理条例》(国令第717号)，涉及我院人类遗传资源的采集、保藏、国际合作、材料出境等行为均需向国家科技部申请行政许可，信息对外提供或开放使用需向国家科技部申请备份备案，通过后方可实施相应活动。临床研究管理部作为全院人类遗传资源管理部门，咨询电话85422851。

在试验（研究）过程中，若变更主要研究者，对临床研究方案、知情同意书等的任何修改，请申请人提交修正案审查申请。

发生严重不良事件，请申请人及时提交严重不良事件报告；紧急报告之后，尽快提交详细的严重不良事件随访报告。

请递交年度和定期跟踪审查报告；当出现任何可能显著影响试验（研究）进行或增加受试者危险的情况时，请申请人及时向伦理审查委员会提交书面报告。

试验（研究）纳入了不符合纳入标准或符合排除标准的受试者，符合中止试验（研究）规定而未让受试者退出试验（研究），给予错误治疗或剂量，给予方案禁止的合并用药等没有遵从方案开展研究的情况；或可能对受试者的权益/健康、以及研究的科学性造成不良影响等违背伦理原则与规范的情况，请申办者/监查员/研究者提交违背方案报告。

申请人暂停或提前终止临床试验（研究），请及时提交暂停/终止试验（研究）报告。

完成临床试验（研究），请申请人提交结题报告。

未经伦理审查批准,不能开展临床研究。

本批件有效期为一年，逾期未实施的，则自行废止。

根据国际医学期刊编辑委员会（ICMJE）要求，所有在人体中和采用取自人体的标本进行的临床研究均应注册。请接到伦理批件的研究者务必在临床研究开始前到中国临床研究注册中心注册，请使用我院公共账号(请发邮件到临床研究管理部邮箱hxlcyjglb@163.com申请，联系电话：85422851)登陆以下网址进行临床研究注册：http://www.chictr.org.cn,临床研究项目注册成功后产生的唯一注册号请及时发送邮件到伦理办公室（huaxilunli@163.com），是伦理跟踪审查的必查项目。

单 位（章）：四川大学华西医院生物医学伦理审查委员会

主任委员（签名）：邓绍林

2020年12月21日
