# Supplementary material for: The optimum parameters and neuroimaging mechanism of repetitive transcranial magnetic stimulation to post-stroke cognitive impairment, a protocol of an orthogonally-designed randomized controlled trial
Source: PLoS One. 2022 Jul 21;17(7):e0271283. doi: 10.1371/journal.pone.0271283 (PMC9302729; doi:10.1371/journal.pone.0271283)
Supplement: S3 File — (DOCX) [file pone.0271283.s003.docx]

Approved by Ethics Committee on Biomedical Research, West China Hospital of Sichuan University

No. 2020(1121)

| Department/Specialty: Department of Rehabilitation Medicine/Rehabilitation Medicine | | | | Project leader Name/title: Li Ling-Xin/attending physician | |
| --- | --- | --- | --- | --- | --- |
| Project name | Study on parameter optimization and neuroimaging of rTMS treating to PSCI | | | | |
| Study protocol | | Version number: V2.0 | | | Version Date: November 23, 2020 |
| Informed consent | | Version number: V1.0 | | | Version Date: October 10, 2020 |
| Recruitment advertisement | | | No | | |
| Review Comments:  1. Researchers' qualifications meet ethical requirements.  2. Basic ethical requirements of the research scheme and informed consent form.  Review results:   ☑Approved 🗆Approved after amending 🗆Review after amending  🗆Not approved 🗆Suspended or terminated the study  Frequency of ongoing reviews:  🗆3 months  🗆6 months  ☑1 year  🗆NA  Please follow the relevant laws, laws and regulations in China (and the biomedical research ethics review method ", etc.) and WMA the CIOMS and octyl speaking is the human body biomedical research international moral guide, follow the ethical review board approval and informed consent to carry out clinical trials (research), protect the health and rights of the subjects.  Please strictly enforce the law of the People's Republic of China into the class of genetic resource management ordinance (no. 717), and our human genetic resources collection, love, international cooperation, material exit all needs to apply to the national Ministry of Science and Technology, the action of administrative license, then you can use the information available or open to the national Ministry of Science and Technology application backup for the record, and through the rear can implement the corresponding activities, The Department of Clinical Research Management, as the department of human genetic resources management of the hospital, tel: 85422851.  In the course of the trial (study), if the principal investigator is changed, any modification to the clinical study protocol, the letter of understanding, etc., please submit the amendment review application.  If a serious adverse event occurs, the applicant should submit a serious adverse event report in time; Detailed follow-up reports of serious adverse events should be submitted as soon as possible after the emergency report.  Please submit annual and periodic follow-up review reports; Applicants are requested to submit written reports to the IRB in a timely manner in the event of any situation that may significantly affect the conduct of the study or increase the risk to the subject.  The study included subjects who did not meet the inclusion criteria or the exclusion criteria, failed to withdraw subjects from the study in accordance with the discontinuation criteria, gave incorrect treatment or dose, and gave combination drugs that were prohibited by the protocol, etc., which did not comply with the protocol; Or the violation of ethical principles and norms, which may have adverse effects on the rights and interests/health of the subjects, as well as the scientific nature of the study, shall be submitted by the sponsor/supervisor/researcher.  If the applicant suspends or discontinues the clinical trial (study) in advance, please submit the discontinue trial (study) report in time.  To complete the clinical trial (study), please submit the final report.  Clinical research cannot be carried out without ethical review and approval.  This approval will be valid for one year. If it is not implemented within the time limit, it will be abolished automatically.  According to the requirements of ICMJE, all clinical studies conducted in and with human specimens should be registered. Investigators who receive ethical approval must register with the Chinese Clinical Research Registry at the beginning of clinical studies. Please use the public account of our hospital (please apply by email to the department of Bed Research hxlcyjglb@163.com, tel: 85422851) and log on to the following website for clinical study note: http://www.chictr.og.cn, the unique registration number generated after the successful registration of the clinical research project, please send an email to the Ethics Office (huaxilunli@163.com) in time, which is a required item for ethics follow-up review.    Units: West China Hospital of Sichuan University  Chairman (Signature) : Shaolin Deng | | | | | |
