# Supplementary material for: The optimum parameters and neuroimaging mechanism of repetitive transcranial magnetic stimulation to post-stroke cognitive impairment, a protocol of an orthogonally-designed randomized controlled trial
Source: PLoS One. 2022 Jul 21;17(7):e0271283. doi: 10.1371/journal.pone.0271283 (PMC9302729; doi:10.1371/journal.pone.0271283)
Supplement: S4 File — (DOC) [file pone.0271283.s004.doc]

内 部 资 料

注 意 保 密

**生物医学伦理研究方案**

**（干预性临床研究）**

重复经颅磁刺激治疗卒中后认知障碍参数优化及神经影像学研究

研究单位：四川大学华西医院

项目负责人（签名）：李凌鑫

承担科室：康复医学科/康复医学系

联系电话：13908198651

组长单位：无

参加单位：无

研究年限：2020年6月－ 2023年12月

（研究开展时间须在伦理批准之后）

版本号：V4.0

版本日期：2020年10月10日

方 案 摘 要

| **研究设计**  **(可多选)** | **□病例对照研究 □队列研究 □横断面研究**  **■随机对照研究 ■应用盲法 □其他：** |
| --- | --- |
| **研究类型**  **（请根据项目类型勾选）** | **（A类：高风险）**  □ 基因编辑研究  □ 细胞治疗研究  □ 植入性医疗器械研究（含3D打印）  □ Ⅲ类临床新技术（安全性、有效性确切，技术难度大、风险高）  □ 特殊人群研究（儿童、孕妇、智力低下者、精神障碍受试者等）  □ 超药物说明书研究（□超适应症 □超给药途径 □超剂量 □超年龄  □超禁忌症 □超人群 □其他，请说明： ）  □ 超器械说明书研究（□超适应症 □使用范围 □超禁忌症 □超人群  □其他，请说明： ）  □ 其他（研究者判定，请说明： ）  **（B类：中风险）**  □ 上市后生物制剂研究（预防用和治疗用）  □ 上市后治疗性疫苗研究  □ 上市后罕见病药物研究  □ Ⅱ类临床新技术（安全性、有效性确切，有一定技术难度，有一定医疗风险和伦理风险）  □ 其他（研究者判定，请说明： ）  **（C类：低风险）**  □ 已上市5年药物研究（包括化药、仿制药等）  □ 已上市器械研究（含AI，影像软件）  ■ Ⅰ类临床新技术（安全性、有效性确切，技术难度低、几乎不存在伦理风险的医疗技术）  □ 其他（传统中医外治疗法，安全性高，技术难度低、几乎不存在伦理风险） |
| **病例总数** | 45 |
| **风险/受益分析** | / |
| **风险判断** | □不大于最小风险 ■大于最小风险  最小风险：指试验中预期风险的可能性和程度不大于日常生活、或进行常规体格检查或心理测试的风险 |
| **研究期限** | 2020 年 6 月 1 日 至 2022 年 12 月 31 日 |

一、研究背景

脑卒中为我国首位致残、致死性疾病[1]。卒中后认知障碍（post-stroke cognitive impairment，PSCI）指在卒中后6个月内出现并达到认知障碍诊断标准的临床综合征，表现为记忆、语言、注意、执行、视空间、运用等一个或多个认知领域功能的下降。据报道，欧洲国家卒中后3个月发生认知功能障碍的发病率高达96%[2]，在我国，PSCI发病率约为55.9%-80.97%[3,4]。研究表明，卒中后发生认知障碍的风险高于无卒中病史者约4-12倍[5]，且其中约6%-32%的PSCI患者会进展为痴呆[6]。卒中后生存患者常因认知障碍影响整体神经功能恢复，延长住院时间，降低生活质量，增加死亡率，是影响脑卒中预后的关键因素[7]，给患者、家庭、社会均带来沉重负担，而长期以来临床实践中却未足够关注和识别卒中后认知功能障碍。在卒中后认知障碍治疗策略中，除了控制危险因素、调节生活方式、认知训练被认为是目前被广泛证实有效的干预措施外，药物治疗方面目前没有一致的证据证实在预防健康老年人认知能力下降方面，任何现有药物的疗效和安全性[8]，因此非药物疗法在卒中后认知障碍临床实践和科学研究中越来越广泛，特别是重复经颅磁刺激疗法(repetitive transcranial magnetic stimulation, rTMS)在防治卒中后认知障碍中显示出一定作用 [9]。然而，现有相关研究提示rTMS治疗卒中后认知障碍的刺激参数、临床疗效和生物学机制总体上仍十分不明确的，例如，一方面有研究表明低频rTMS[10]而非高频rTMS[11]对卒中后认知障碍有改善作用，而另一些研究又提示高频对卒中后认知功能有明显改善作用[12]而低频rTMS对卒中后认知功能的改善作用尚不能肯定[13]。另一方面对于rTMS的刺激部位的选择在诸多现有研究中也混杂不一，左侧颞顶叶皮层(temporoparietal cortex, TPC)、后顶叶皮层（posterior parietal cortex, PPC）、左或右侧背外侧前额叶皮层(dorsolateral prefrontal cortex, DLPFC)、前颞叶(anterior temporal lobes, ATL)、左前下额叶皮层(left anterior inferior frontal cortex，LAIFC)等不同皮层部位的rTMS刺激，均在相应研究中报道有改善卒中后不同认知域功能的作用[14,15]。综上可以看出，rTMS技术因不同刺激频率和不同刺激部位而在卒中后认知障碍的疗效及最佳参数的确定性上的混杂不一给临床实践带来巨大困扰。由此可见，既往相关研究均仅从某一刺激频率或某一刺激部位的单一参数因素来观察rTMS治疗卒中后认知障碍的临床疗效和安全性的研究方法存在根本缺陷，在此基础上对其作用机制的探索和论证显得更为乏力。基于上述现状，本研究创新性借鉴最优化研究中的正交设计思路，复习相关文献，选择rTMS技术中刺激部位、刺激频率、刺激强度、刺激个数四个重要参数，选择临床中最常用的DLPFC、IFG、TPC三个刺激部位；5Hz、10Hz、20Hz三种刺激频率；90%RMT，100%RMT，110%RMT三种刺激强度和1000个，1500个，2000个三种刺激个数，运用四因素三水平的L_9_(3^4^)正交设计表，优化rTMS刺激参数，并运用脑功能磁共振成像技术，从神经影像学角探索重复经颅磁刺激治疗卒中后认知障碍的可能机制，对rTMS临床实践具有十分重要的指导意义。

二、研究目的

1. 主要目的：优化rTMS技术治疗卒中后认知障碍的刺激参数并评价其临床疗效及安全性。

2. 次要目的：探索rTMS改善卒中后认知功能的神经影像学机制。

三、研究设计、方法与研究步骤

1. 研究设计

本研究在循证医学临床研究试验报告统一标准2010年声明(Consolidated Standards of Reporting Trials, CONSORT 2010 statement)及非药物疗法临床研究试验报告统一标准2017更新版（CONSORT Statement for Randomized Trials of Nonpharmacologic Treatments: A 2017 Update，CONSORT statement of NPT 2017）前提下，根据优化rTMS刺激参数的研究目的，运用正交设计研究方法设计随机对照临床试验。本研究规定刺激个数、刺激频率和刺激部位四因素各自三个不同水平，见表1。用SPSS 22. 0软件随机生成 L9(33)正交表(随机种子数 seed = 100)，将试验随机分为9组，见表2：


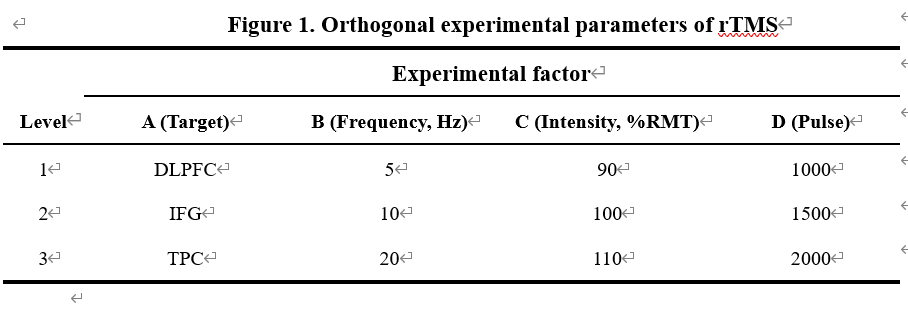


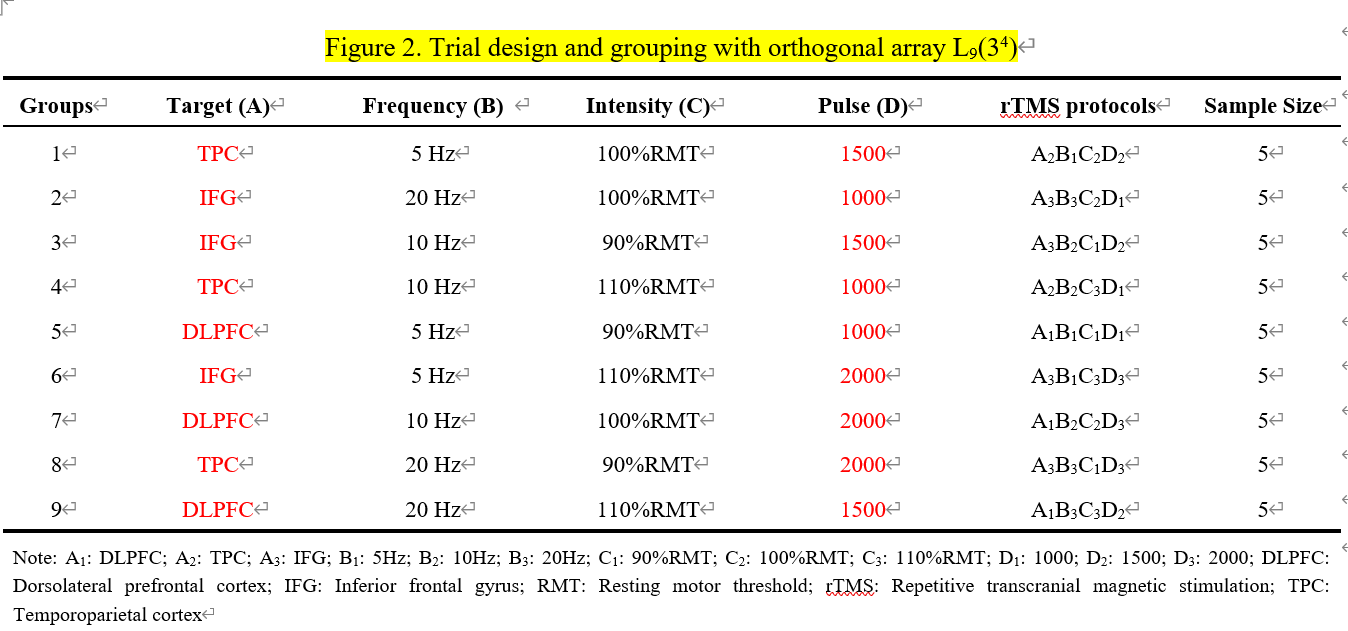


1. 研究方法

本研究运用正交设计方法，选择重复经颅磁刺激技术刺激部位（背外侧前额叶皮层、额下回皮层、颞顶叶皮层），刺激频率（5Hz、10Hz、20Hz），刺激强度（90%RMT，100%RMT，110%RMT）和刺激个数（1000、1500、2000）四个参数，每种参数设计三种不同刺激水平，运用四因素三水平的L_9_(3^4^)正交设计研究，组合为9个试验组，从认知功能、个体活动能力、社会参与能力及rTMS不良反应等临床疗效和安全性方面进行重复经颅磁刺激治疗卒中后认知障碍的刺激参数最优化筛选研究，并运用脑功能磁共振成像检测技术，从神经影像学角探索重复经颅磁刺激治疗卒中后认知障碍的生物学机制。

3. 研究步骤


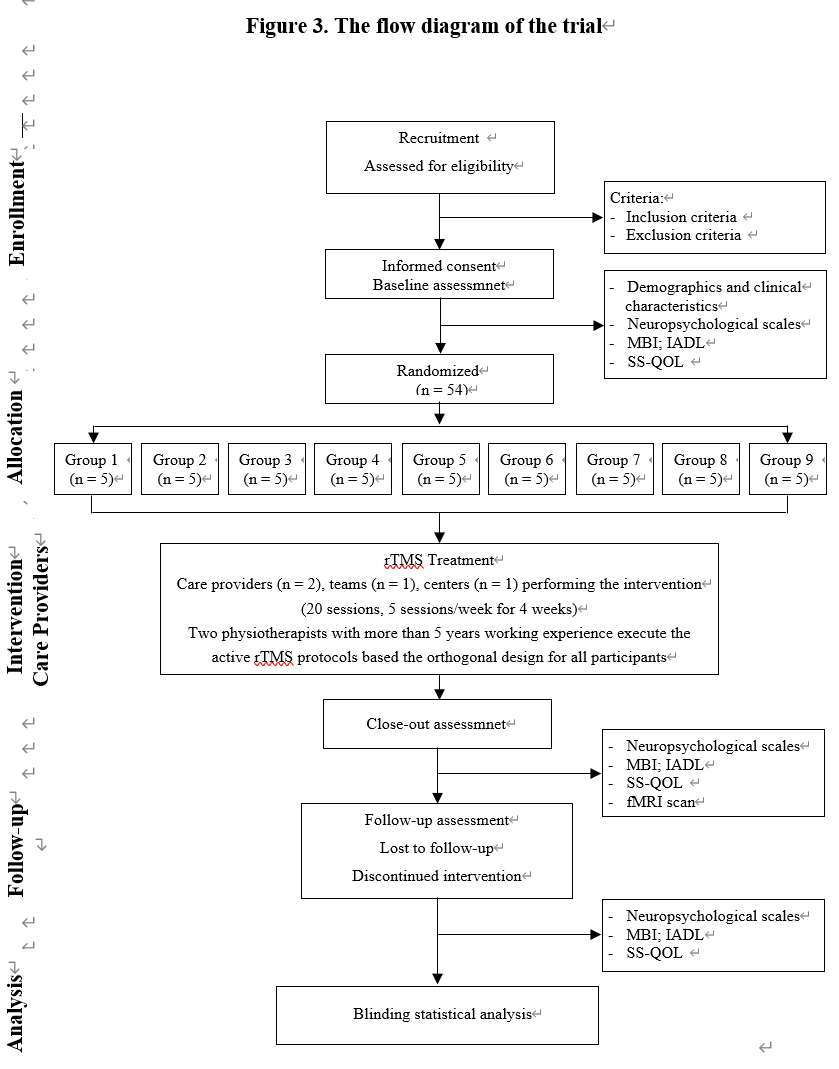


四、病例选择

1. 入选标准

(1)符合缺血性脑卒中、自发性脑出血诊断标准；

(2)依据MMSE/MoCA认知测验（至少评估4项认知域：执行功能/注意力、记忆、语言能力、视空间能力），至少≥1项认知域受损。具体分值：MMSE：文盲≤17分；小学≤20分；中学或以上≤24分； MoCA：＜24分。

(3)日常生活能力受损非继发于血管事件的运动/感觉功能缺损所致。

(4)年龄、性别、病程：性别不限，年龄18-75岁；右利手；首次发病；病程：大于90天，小于180天；无严重失语；发病前认知功能正常。

(5)签署知情同意书者。

2. 排除标准

(1)原发性或继发性神经疾病所致的认知障碍，如正常颅压脑积水、额颞叶痴呆、帕金森病、多发性硬化症、脑炎和谵妄;

(2)由抑郁症、精神分裂症、双相情感障碍、精神病性障碍、维生素D缺乏、中毒或其他系统性疾病引起的认知障碍;

(3)认知损伤前3个月内存在药物/酒精滥用/依赖。

(4)rTMS治疗禁忌症，如癫痫患者、孕妇或哺乳期妇女，或使用金属或电植入设备(如深部脑刺激器、脑室腹腔分流器、动脉瘤夹、起搏器、耳蜗、头皮上的手术钉)。

(5)MRI禁忌症(如金属植入物或幽闭恐惧症)

(5)同时参与其他药理学或非药理学治疗研究。

3. 终止研究标准

(1)受试者主动提出退出者；

(2)研究过程中出现严重不良反应而不宜继续参加本研究者；

(3)研究过程中出现严重并发症或出现病情恶化，需采取紧急措施者;

(4)研究人员应详细记录退出研究的原因及时间。

1. 可供选择的其他诊疗方法

可替代的中医康复方案：针刺疗法；推拿疗法；中药疗法。

可替代的西医康复方案：运动疗法；物理因子疗法；康复支具疗法等。

六、检测项目与检测时点

1 主要结局指标

(1)认知神经心理量表：MoCA,TMT

2 次要结局指标

(1)个体活动能力指标：MBI-C、LIADL

(2)社会参与能力指标：SS-QOL-12

(3)安全性指标：血常规、肝功能、肾功能、凝血功能；rTMS相关的不良反应，如头痛、恶心、癫痫、不良情绪、肢体无力、耳鸣、疲劳、失眠等。

(4)机制指标：fMRI

检测时点

1. 基线检测：受试者签署知情同意书入组之后，治疗干预前由两名专门研究人员独立进行结局指标基线检测。
2. 治疗结束检测：所有的4周治疗干预结束后即刻由相同研究人员独立进行结局指标检测。
3. 随访检测：时点为疗程结束后3个月随访，随访地点，检测方式、内容和基线及治疗结束时检测一致。
4. 检测地点

在四川大学华西医院康复医学科的住院患者。全部研究在康复科住院部、康复治疗室、相关辅助检查室完成。

七、疗效评定标准

1. 临床疗效标准，参考卒中后认知障碍管理专家共识 2017 确定

痊愈：MMSE：文盲>17 分，小学>20 分，中学或以上>24 分或 MoCA：>24 分

显效：MMSE 或 MoCA 提高≥5 分。

有效：MMSE 或 MoCA 提高 1-4 分。

无效：MMSE 或 MoCA 评分无变化或分值降低

1. 综合疗效标准

痊愈：功能正常，生活完全独立

显效：功能轻度障碍，生活基本自理

有效：功能中重度障碍，生活明显或严重依赖

无效：生活完全依赖

1. 安全性评定标准

安全：无与本疗法肯定相关或有与本疗法肯定无关的临床症状与体征、生命体征异常、或实验室检查异常发生。

可能安全：有与本疗法很可能/可能相关的临床与体征、生命体征异常、或实验室检查异常发生。

不安全：有与本疗法肯定相关的临床与体征、生命体征异常、或实验室检查异常发生。

八、不良事件的观察、记录和处置

不良事件定义：

与经颅磁刺激相关的不良事件包括：

1）头痛

2）恶心

3）疼痛

4）癫痫

不良事件处理：

该研究以脑卒中患者为受试者研究，其本身及参加研究过程中均存在一定风险，针对风险我们进行了如下应急预案：

1）所有受试者均为住院患者或健康人，具有完善的医治护团队保证意外发生时能得到及时救治。

2）一旦发生如休克、高血压危象、癫痫持续状态、跌倒等不良事件后，首先向医疗组长汇报，按院内不良事件上报，同时协助医疗组按照标准化SOP流程进行处理。

3） 电话或门诊随访，至不良反应相关症状体征完全消失。

不良事件记录：

所有不良事件均应按不良事件报告表记录，报告，处理，随访。

九、研究的质量控制与质量保证

本研究过程中，将由课题组指派的临床监查员定期进行现场监督查访，以保证研究方案的所有内容都得到严格遵守和填写研究资料的正确性。

1. 对专业人员进行课题实施方案的培训，要求熟练掌握干预方案的操作、实施、指标的观察与检测等。
2. 针对进行临床研究的特点与难度，全面分析可能出现的混杂因素，以减少研究结果的偏倚。明确主研人员分工，要求各中心严格遵守和执行顶层设计的技术路线，认真、按时完成课题任务。
3. 各病区应相对固定研究人员，必须经过统一培训，避免在课题执行中更换，以保证研究质量。
4. 严格执行治疗方法的操作规范、适应症、禁忌症及用药原则。
5. 严格按照临床工作手册和填表说明填写CRF表，记录观察病历详细资料。CRF表录入要求认真、完整，并将相应的检查报告附后，原始数据不得随意更改，如有更改，必须详细注明原因及更改者签名。
6. 实验室检查的异常判断标准，以检查单位的正常参考范围为准。
7. 在研究方案的实施中保证患者的依从性。
8. 针对可能发生的脱落，积极采取措施，控制病例脱落率在l5％以内。
9. 监查员定期检查，抽样核对原始资料与录入数据的一致性。对不按期完成任务者采取措施，及时淘汰和处罚。
10. 数理统计人员早期介入，并对课题运行全程予以监督。数据管理中心要及时汇总各中心的原始数据进行存档。原始数据录入要求真实、完整，并将相应的检查报告附后，如有更改必须详细注明变更原因、时间，并需更改者签名。

十、数据安全监查

临床研究将根据风险大小制定相应的数据安全监察计划。所有不良事件均详细记录，恰当处理并追踪直到妥善解决或病情稳定，按照规定及时向伦理审查委员会、主管部门、申办者和药品监督管理部门报告严重不良事件与非预期事件等；主要研究者定期对所有不良事件进行累积性回顾，必要时召开研究者会议评估研究的风险与受益；双盲试验必要时可以进行紧急揭盲，以确保受试者安全与权益。

十一、统计学处理

所有统计分析用SPSS22.0软件完成。对连续性变量，在基线、干预结束、随访时点分别用两独立样本t检验或Wilcoxon秩和检验进行组间比较，用配对t检验或Wilcoxon 配对符号秩和检验进行组内比较。对于连续变量的变化量（从基线至干预结束，或从基线到随访时点），用协方差进行组间比较。对非连续变量，用Cochran-Mantel-Haenszel卡方检验或Fisher确切检验进行组间比较，用McNemar卡方检验进行组内比较。双侧P < 0.05，具有统计学意义。

十二、临床研究伦理原则与要求

临床研究将遵循世界医学大会《赫尔辛基宣言》和中华人民共和国国家卫生和计划生育委员会《涉及人的生物医学研究伦理审查办法》等相关规定，具体落实知情同意，保护隐私，研究免费与补偿，控制风险，特殊受试者保护和研究相关损害的赔偿原则与要求。在研究开始之前，由伦理审查委员会批准该试验方案后才实施临床研究。每一位受试者入选本研究前，研究者有责任向受试者或/和其法定代理人完整、全面地介绍本研究的目的、程序和可能的风险，并签署书面知情同意书，应让受试者知道他们参加临床研究完全是自愿的，他们可以拒绝参加或在试验的任何阶段随时退出本研究而不会受到歧视和报复，其医疗待遇与权益不受影响。知情同意书应作为临床研究文件保留备查，切实保护受试者的个人隐私与数据机密性。

十三、研究进度

2020年6月-2020年9月：进一步完成文献查阅、资料检索，充分完成试验准备工作，完成试验方案、SOP制度，CRF表等资料；申请伦理、注册；协调影像检测事务

2020年10月-2020年12月：完成预试验，完善各项试验条件

2021-年1月-2022年3月：进行临床试验，完成正式临床实验

2022年4月-2022年6月：整理数据，撰写论文，并总结资料，结题。

十四、参加人员

| **姓名** | **职称/专业** | **任务** | **GCP培训证书** |
| --- | --- | --- | --- |
| 李凌鑫 | 中级-主治医师 | 课题负责，设计实施、结题、总体安排，治疗干预 | WZ012020009899 |
| 王婷婷 | 博士 | 病例入组，临床管理，知情告知 |  |
| 张永刚 | 本科 | 治疗干预 |  |
| 杨羲瑞 | 本科 | 量表评估 |  |

1. 主要参考文献

[1].王陇德. 中国脑卒中防治报告[R]. 北京：中国协和医科大学出版社，2016.

[2].Sun JH, Tan L, Yu JT. Post-stroke cognitive impairment: epidemiology, mechanisms and management[J]. Ann Transl Med, 2014, 2: 80.

[3].曲艳吉, 卓琳, 詹思延.中国脑卒中后认知障碍流行病学特征的系统评价[J]. 中华老年心脑血管病杂志,2013,15(12):1294-1301.

[4].Qu Y, Zhuo L, Li N, et al. Prevalence of poststroke cognitive impairment in China: a community based, cross-sectional study[J]. PLoS ONE, 2015, 10: e0122864.

[5].Renjen PN, Gauba C, Chaudhari D. Cognitive impairment after stroke[J]. Cureus, 2015, 7: e335.

[6].Pasi M, Poggesi A, Salvadori E, et al. Post-stroke dementia and cognitive impairment[J]. Front Neurol Neurosci,2012,30:65-69.

[7].Danovska M, Stamenov B, Alexandrova M, et al. Post-stroke cognitive impairment phenomenology and prognostic factors[J]. Journal of IMAB, 2012, 18: 290-297.

[8].中国痴呆与认知障碍诊治指南写作组. 2018中国痴呆与认知障碍诊治指南(五)：轻度认知障碍的诊断与治疗. 中华医学杂志，2018，98(17):1294-1301.

[9].徐菁菁,曹忠耀,张清华.重复经颅磁刺激联合认知训练治疗非痴呆型血管性认知障碍疗效观察[J].现代中西医结合杂志,2018,27(34):3768-3771.

[10]. Sebastianelli L, Versace V, Martignago S, Brigo F, Trinka E, Saltuari L, Nardone R. Low-frequency rTMS of the unaffected hemisphere in stroke patients: A systematic review.Acta Neurol Scand. 2017 Dec;136(6):585-605.

[11].Guse B, Falkai P, Wobrock T. Cognitive effects of high-frequency repetitive transcranial magnetic stimulation: a systematic review. J Neural Transm. 2010;117:105-122.

[12]. Ma Q, Geng Y, Wang HL, Han B, Wang YY, Li XL, Wang L, Wang MW. High Frequency Repetitive Transcranial Magnetic Stimulation Alleviates Cognitive Impairment and Modulates Hippocampal Synaptic Structural Plasticity in Aged Mice. Front Aging Neurosci. 2019;11:235. doi: 10.3389/fnagi.2019.00235. eCollection 2019.

[13]. Lage C, Wiles K, Shergill SS, Tracy DK. A systematic review of the effects of low-frequency repetitive transcranial magnetic stimulation on cognition.J Neural Transm (Vienna). 2016;123(12):1479-1490.

[14].Pobric, G., E. Jefferies and M.A. Ralph, Anterior temporal lobes mediate semantic representation: mimicking semantic dementia by using rTMS in normal participants. Proc Natl Acad Sci U S A, 2007. 104(50): 20137-20141.

[15].Gough PM, Nobre AC, Devlin JT. Dissociating linguistic processes in the left inferior frontal cortex with transcranial magnetic stimulation. J Neurosci. 2005;25:8010-8016.
