# Supplementary material for: The optimum parameters and neuroimaging mechanism of repetitive transcranial magnetic stimulation to post-stroke cognitive impairment, a protocol of an orthogonally-designed randomized controlled trial
Source: PLoS One. 2022 Jul 21;17(7):e0271283. doi: 10.1371/journal.pone.0271283 (PMC9302729; doi:10.1371/journal.pone.0271283)
Supplement: S5 File — (DOC) [file pone.0271283.s005.doc]

**Biomedical ethics research program**

**(Interventional clinical study)**

The study of parameter optimization and neuroimaging of rTMS in PSCI patients

Setting: West China Hospital of Sichuan University

Project Leader (signature): Li Lingxin

Department: Department of Rehabilitation Medicine

TEL: 13908198651

Group leader: None

Participating unit: None

Study period: June 2020 to December 2023

(The study shall be conducted after ethical approval)

Version: V4.0

Version Date: October 10, 2020

**Protocol Abstract**

| **Study design**  **(Multiple options)** | **□ Case control study □ Cohort study □ Cross-sectional study**  **■ Randomized controlled study ■ Blind Method □ Others:** |
| --- | --- |
| **Research type**  **(Please check according to project type)** | **(Category A: High risk)**  □ Gene editing research  □ Cell therapy research  □ Research on implantable medical devices (including 3D printing)  □ Class ⅲ new clinical technology (safety, effectiveness, technical difficulty, high risk)  □ Studies on special populations (children, pregnant women, persons with mental retardation, subjects with mental disorders, etc.)  □ Out of label study (□ out of indication □ out of route □ out of dose □ out of age  □ Excess contraindications □ Excess population □ Others, please specify:)  □ Study on specifications of ultra - device (□ ultra - indications □ Use range □ ultra - contraindications □ Ultra - crowd  □ Others, please specify:)  □ Others (as determined by researchers, please specify:)  **(Category B: medium risk)**  Research on post-marketing biologics (preventive and therapeutic)  □ Research on post-marketing therapeutic vaccines  □ Research on rare disease drugs after marketing  □ Class ⅱ clinical new technology (with definite safety and effectiveness, certain technical difficulty, certain medical and ethical risks)  □ Others (as determined by researchers, please specify:)  **(Category C: Low risk)**  □ Has been on the market for 5 years (including chemical drugs, generic drugs, etc.)  □ Research on marketed devices (including AI and imaging software)  ■ Class ⅰ new clinical technologies (medical technologies with proven safety and efficacy, low technical difficulty and almost no ethical risk)  □ Others (External treatment of traditional Chinese medicine, with high safety, low technical difficulty and almost no ethical risk) |
| **Sample** | 45 |
| **Risk/Benefit analysis** | / |
| **Risk judgment** | □ Greater than minimum risk ■ Greater than minimum risk  Minimum risk: the likelihood and extent of the risk expected in the trial is not greater than the risk of daily life or of routine physical examination or psychological testing |
| **Study Timeline** | From 1 June 2020 to 31 December 2023 |

**I. Background**

Stroke is the first disabling and fatal disease in China [1]. Post-stroke cognitive impairment (PSCI) refers to the clinical syndrome that appears within 6 months after stroke and meets the diagnostic criteria of cognitive impairment. It is manifested as the decline of functions in one or more cognitive areas such as memory, language, attention, execution, visual space and application. It has been reported that the incidence of cognitive dysfunction 3 months after stroke in European countries is as high as 96%[2], while in China, the incidence of PSCI is about 55.9%-80.97%[3,4]. Studies have shown that the risk of cognitive impairment after stroke is about 4-12 times higher than that of those without stroke history [5], and about 6%-32% of PSCI patients will progress to dementia [6]. Survival after stroke patients with cognitive impairment often affect the whole neural functional recovery, extend the length of hospital stay, lower the quality of life, increase mortality, is the key factor affecting the prognosis of stroke [7], to patients, families and society bring heavy burden, and the clinical practice for a long time is not enough attention and recognition of cognitive dysfunction after stroke. In cognitive impairment after stroke treatment strategies, in addition to the control of risk factors, adjust the way of life, cognitive training is considered to be currently widely proved to effective intervention measures, there is no consistent evidence of drug treatment in preventing cognitive decline in the elderly health, the efficacy and safety of any existing drugs [8], Therefore, non-drug therapy has been increasingly widely used in the clinical practice and scientific research of post-stroke cognitive impairment, especially repetitive transcranial magnetic stimulation (rTMS), which has shown a role in the prevention and treatment of post-stroke cognitive impairment [9]. However, existing studies suggest that the stimulus parameters, clinical efficacy and biological mechanism of rTMS in the treatment of post-stroke cognitive impairment are still very unclear. For example, on the one hand, some studies have shown that low frequency rTMS[10] but not high frequency rTMS[11] can improve post-stroke cognitive impairment. Other studies suggest that high frequency can significantly improve the cognitive function after stroke [12], while the effect of low frequency rTMS on the cognitive function after stroke cannot be confirmed [13]. On the other hand, the selection of stimulation sites for rTMS is also mixed in many existing studies. Temporoparietal cortex (TPC), posterior parietal cortex (PPC), left or right dorsolateral prefrontal cortex, RTMS stimulation in DLPFC, ATL, LAIFC and other cortical regions of anterior temporal cortex Both have been reported to improve the function of different cognitive domains after stroke in corresponding studies [14,15]. In summary, the mixed efficacy and the certainty of optimal parameters of rTMS technique in post-stroke cognitive impairment due to different stimulation frequencies and stimulation sites have brought great confusion to clinical practice. It can be seen that previous relevant studies only observe the clinical efficacy and safety of rTMS in the treatment of post-stroke cognitive impairment from a single parameter factor of a certain stimulus frequency or a certain stimulus site. There are fundamental defects in the research methods, and on this basis, the exploration and demonstration of its mechanism of action are more weak. Based on the above situation, this study innovatively referred to the orthogonal design idea of optimization research, reviewed relevant literature, and selected four important parameters of rTMS technology, including stimulation site, stimulation frequency, stimulation intensity and stimulation number, and three stimulation sites, DLPFC, IFG and TPC, which were most commonly used in clinical practice. 5Hz, 10Hz, 20Hz three stimulus frequency; Three kinds of stimulation intensity of 90%RMT, 100%RMT and 110%RMT and three kinds of stimulation number of 1000, 1500 and 2000 were used to optimize rTMS stimulation parameters by L9(34) orthogonal design table with four factors and three levels. Functional magnetic resonance imaging (FMRI) was used. Exploring the possible mechanism of repetitive TRANSCranial magnetic stimulation (rTMS) in the treatment of cognitive impairment after stroke from the neuroimaging Angle is of great guiding significance to the clinical practice of rTMS.

**II. Research objectives**

1. Main objective: To optimize the stimulation parameters of rTMS in the treatment of post-stroke cognitive impairment and evaluate its clinical efficacy and safety.

2. Secondary objective: To explore the neuroimaging mechanism of rTMS in improving cognitive function after stroke.

**III. Research design, methods and procedures**

1. Research design

This study is based on the Consolidated Standards of Reporting Trials 2010 Statement (Consolidated Standards of Reporting Trials, CONSORT Statement for Randomized Trials of Nonpharmacologic Treatments CONSORT statement for Randomized Trials of Nonpharmacologic Treatments A 2017 Update, CONSORT Statement of NPT 2017) requires that A randomized controlled clinical trial be designed using orthogonal design research method for the purpose of optimizing stimulation parameters of rTMS. In this study, the number of stimuli, the frequency of stimuli and the location of stimuli were determined at three different levels respectively, as shown in Figure 1. SPSS 22.0 software was used to randomly generate L9(34) orthogonal table (random seed number = 100), and the experiment was randomly divided into 9 groups, as shown in Figure 2.


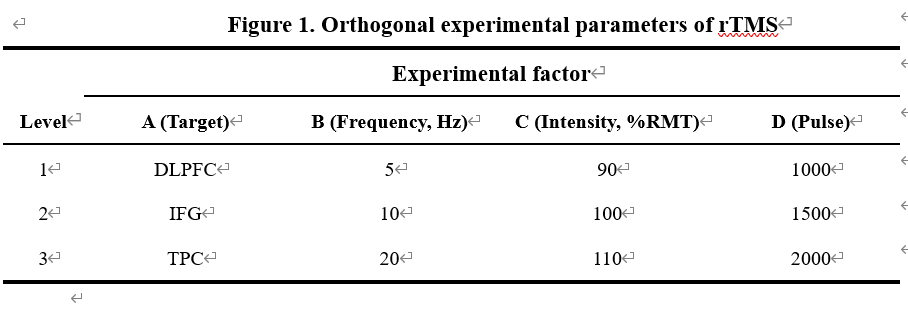


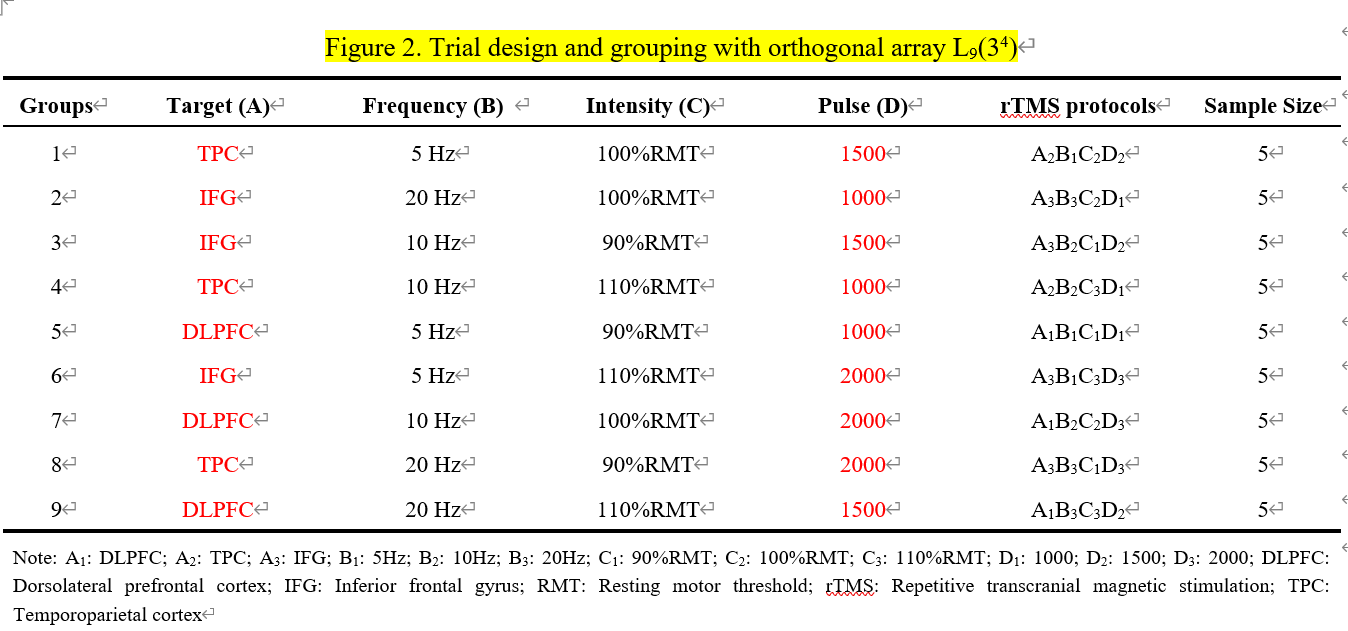


2.Methods

In this study, orthogonal design method was used to select the stimulation sites (dorsolateral prefrontal cortex, inferior frontal gyrus cortex, temporal parietal cortex), stimulation frequency (5Hz, 10Hz, 20Hz), stimulation intensity (90%RMT, 100%RMT, 110%RMT) and stimulus number (1000, 1500, 2000). Three different stimulus levels were designed for each parameter. L9(34) orthogonal design with four factors and three levels was used to study, and 9 experimental groups were combined. From cognitive function, individual activities, social participation and rTMS adverse reactions such as clinical curative effect and safety of repetitive transcranial magnetic stimulation treatment of cognitive impairment after stroke stimulation parameters optimization screening study, and using brain functional magnetic resonance imaging detection technology, from the Angle of neuroimaging to explore repetitive transcranial magnetic stimulation biological mechanisms for the treatment of cognitive impairment after stroke.

3. Flow chart of the trial


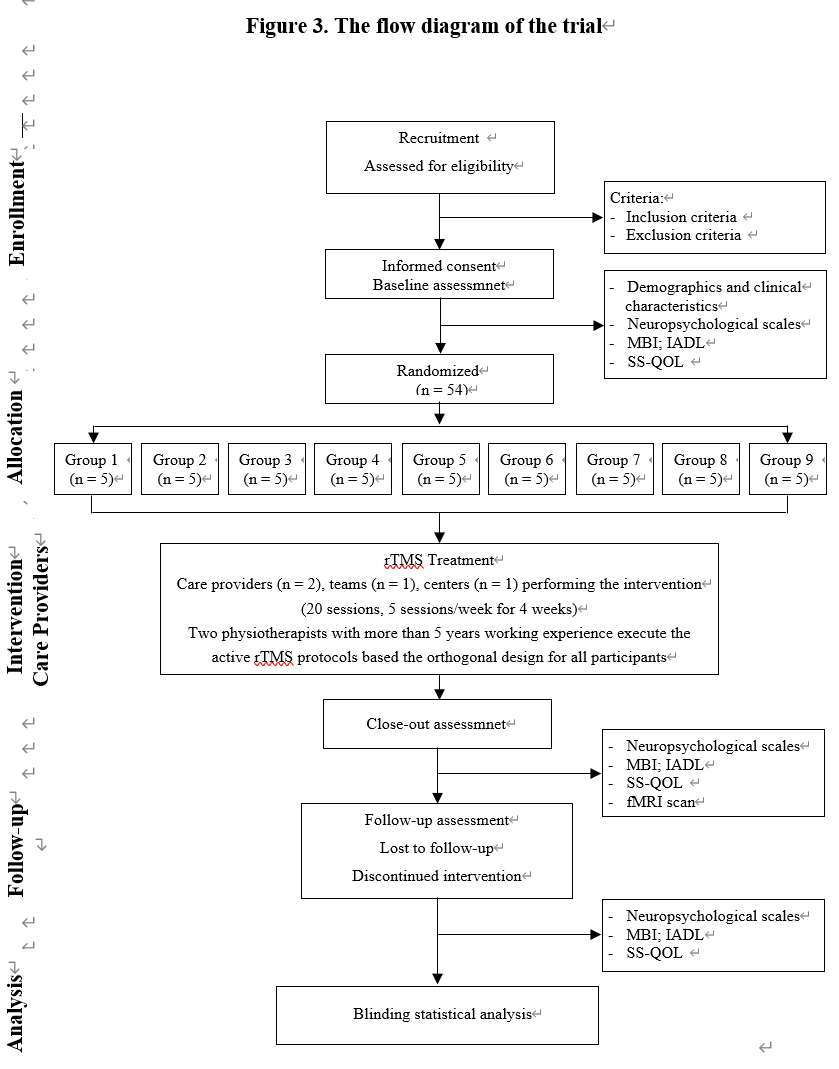


**IV. Patients**

1. Inclusion criteria

(1) Meet the diagnostic criteria of ischemic stroke and spontaneous cerebral hemorrhage;

(2) Impairment of at least 1 cognitive domain according to MMSE/MoCA cognitive test (at least 4 cognitive domains: executive function/attention, memory, language ability, visuospatial ability). Specific score: MMSE: Illiteracy ≤17 points; Primary school ≤20 points; Middle school or above ≤24 points; MoCA: < 24 points.

(3) Impairment of daily living is not caused by motor/sensory deficits secondary to vascular events.

(4) Age, sex and course of disease: no gender limitation, age 18-75; Right-handed. First onset; Duration: more than 90 days, less than 180 days; No severe aphasia; Cognitive function was normal before onset.

(5) The informed consent is signed.

2. Exclusion criteria

(1) Cognitive impairment due to primary or secondary neurological disorders, such as normal cranial pressure hydrocephalus, frontotemporal dementia, Parkinson's disease, multiple sclerosis, encephalitis, and delirium;

(2) cognitive impairment caused by depression, schizophrenia, bipolar disorder, psychotic disorder, vitamin D deficiency, poisoning, or other systemic diseases;

(3) Drug/alcohol abuse/dependence within 3 months prior to cognitive impairment.

(4) Contraindications for rTMS treatment, such as patients with epilepsy, pregnant or lactating women, or the use of metal or electrical implant devices (such as deep brain stimulator, ventriculoperitoneal shunt, aneurysm clip, pacemaker, cochlea, surgical nail on the scalp).

(5)MRI contraindications (such as metal implants or claustrophobia)

(6) Participate in other pharmacological or non-pharmacological treatment studies at the same time.

3. Termination criteria

(1) Subjects who voluntarily withdraw;

(2) If serious adverse reactions occur during the study, it is inappropriate to continue to participate in the study;

(3) Serious complications or deterioration of the condition occur during the study and emergency measures need to be taken;

(4) Researchers should record the reasons and time of withdrawal in detail.

**V. Alternative diagnosis and treatment methods**

Alternative TCM rehabilitation programs: Acupuncture therapy; Massage therapy; Chinese medicine therapy.

Alternative Rehabilitation programs in Western medicine: Exercise therapy; Physical factor therapy; Rehabilitation bracing therapy, etc.

**VI. Test items and test time**

1.Main outcome measures

(1) Cognitive neuropsychological scale: MoCA,TMT

2. secondary outcome measures

(1) Individual activity indicators: MBI-C and LIADL

(2) Indicators of social participation ability: SS-QOL-12

(3) Safety indicators: blood routine, liver function, kidney function, coagulation function; RTMS related adverse reactions, such as headache, nausea, epilepsy, bad mood, limb weakness, tinnitus, fatigue, insomnia, etc.

(4) Mechanism indicators: fMRI

3. Detection point

(1) Baseline testing: After subjects signed informed consent and enrolled, baseline testing of outcome indicators was independently conducted by two specialized researchers before treatment intervention.

(2) End-of-treatment testing: All 4-week intervention outcomes were tested independently by the same researchers immediately after the intervention.

(3) Follow-up test: the follow-up time was 3 months after the end of the treatment course. The follow-up location, test method and content were consistent with baseline and the test at the end of the treatment.

(4) Test site

Inpatients in department of Rehabilitation Medicine, West China Hospital of Sichuan University. All the studies were completed in the rehabilitation inpatient department, rehabilitation treatment room and related auxiliary examination room.

**VII. Evaluation criteria for curative effect**

(1) Clinical efficacy criteria (determined by referring to expert consensus on post-stroke cognitive impairment management 2017)

Cured: MMSE: Illiterate >17, elementary >20, middle school or above >24 or MoCA: >24

Significant effect: MMSE or MoCA increased by ≥5 points.

Effective: Increase by 1-4 points in MMSE or MoCA.

Invalid: No change or decrease in MMSE or MoCA score

(2) Comprehensive efficacy criteria

Cured: Functioning normally and living completely independently

Significant effect: Mild functional impairment, basic self-care

Effective: Moderate to severe functional impairment, obvious or severe dependence on life

Ineffective: Life completely dependent

(3) Safety assessment standards

Safety: No clinical symptoms and signs, abnormal vital signs, or abnormal laboratory tests are positively related to or unrelated to this therapy.

May be safe: Abnormalities in clinical and physical signs, vital signs, or laboratory tests are likely/likely to be associated with the treatment.

Unsafe: Abnormalities in clinical and physical signs, vital signs, or laboratory tests that are positively associated with the treatment.

**VIII. Observation, recording and disposal of adverse events**

1. Adverse events associated with TMS include:

(1) Headache

(2) Nausea

(3) Pain

(4) Epilepsy

2. Adverse event management:

In this study, stroke patients were selected as subjects, and there were certain risks both in themselves and during the study. For these risks, we made the following emergency plans:

(1) All subjects are hospitalized patients or healthy people, and have a complete medical care team to ensure timely treatment when accidents occur.

(2) Once the occurrence of adverse events such as shock, hypertensive crisis, epileptic state, fall and other adverse events, first report to the medical team leader, reported as hospital adverse events, and assist the medical team to handle according to the standardized SOP process.

(3) Telephone or outpatient follow-up until the symptoms and signs related to adverse reactions completely disappeared.

3. Adverse event Records:

All adverse events should be recorded, reported, handled, and followed up in accordance with the Adverse Event Report Form.

**IX. Research quality control and quality assurance**

In the process of this study, clinical inspectors designated by the research group will regularly conduct on-site supervision and visits to ensure that all contents of the research plan are strictly observed and the research materials are correct.

(1) Provide training on project implementation plans for professionals, who are required to master the operation, implementation of intervention plans, observation and detection of indicators, etc.

(2) According to the characteristics and difficulty of clinical studies, possible confounding factors were comprehensively analyzed to reduce the bias of research results. The division of labor of principal researchers was clarified, and all centers were required to strictly observe and implement the technical route of top-level design, and complete the task conscientiously and on time.

(3) Researchers in each ward should be relatively fixed, and must undergo unified training to avoid replacement during project implementation, so as to ensure research quality.

(4) Strictly implement the operation specifications, indications, contraindications and medication principles of the treatment methods.

(5) Fill in the CRF form strictly in accordance with the clinical work manual and form-filling instructions, and record the details of medical records observed. CRF table entry should be careful and complete, and the corresponding inspection report should be attached. The original data should not be changed at will. If there is any change, the reason and the signature of the changer should be specified in detail.

(6) Abnormal judgment criteria of laboratory inspection shall be subject to the normal reference range of the inspection unit.

(7) Ensure patient compliance during the implementation of the study protocol.

(8) For possible shedding, take active measures to control the case shedding rate within l5%.

(9) Inspectors regularly check and sample to check the consistency of original data and input data. Take measures to eliminate and punish those who fail to complete the task on time.

(10) Mathematical statisticians intervened early and supervised the whole operation of the project. The data management center shall timely summarize the original data of each center for archiving. The original data entry shall be true and complete, and the corresponding inspection report shall be attached. If there is any change, the reason and time of change shall be specified in detail, and the signature of the person who changes shall be signed.

**X. Data security supervision**

The clinical study will develop a data security monitoring plan based on the risk. All adverse events are recorded in detail, properly handled and tracked until they are properly resolved or the condition is stable, and serious adverse events and unexpected events are reported to the ETHICS Review Committee, competent authorities, sponsors and drug regulatory authorities in a timely manner as required; The principal investigator periodically conducted cumulative reviews of all adverse events, and convened investigator meetings when necessary to assess the risks and benefits of the study. In double-blind trials, emergency unblinding can be carried out when necessary to ensure the safety and rights of subjects.

**XI. Statistical analysis**

All statistical analysis was performed by SPSS22.0 software. For continuous variables, inter-group comparisons were performed by two independent samples t-test or Wilcoxon rank sum test at baseline, end of intervention, and follow-up, respectively, and intra-group comparisons were performed by paired T-test or Wilcoxon paired signed rank sum test. The amount of change in continuous variables (from baseline to end of intervention, or from baseline to point of follow-up) was compared between groups using covariance. For discontinuous variables, cochran-Mantel-Haenszel chi-square test or Fisher's exact test was used for inter-group comparison and McNemar chi-square test was used for intra-group comparison. P < 0.05 on both sides, which was statistically significant.

**XII. Ethical principles and requirements of clinical research**

Clinical research will follow the world medical conference the declaration of Helsinki and the national health and family planning commission of the People's Republic of China "relating to the people of biomedical research ethics review method" and other relevant provisions, the concrete implementation of informed consent, privacy, free research and compensation, risk control, special subjects related damage compensation principle and requirements of protecting and researching. Prior to the start of the study, the trial protocol will be approved by the ethics review Committee before clinical study. Before each subject is enrolled in this study, the investigator is responsible for providing the subject or his/her legal representative with a complete and comprehensive introduction of the purpose, procedure and possible risks of this study, and signing a written informed consent form. The subject should be informed that his/her participation in the clinical study is completely voluntary. They can refuse to participate in the study or withdraw from the study at any stage of the study without discrimination or retaliation, and their medical treatment and rights and interests will not be affected. Informed consent forms should be kept as clinical research documents for future reference to effectively protect the personal privacy and data confidentiality of subjects.

**XIII. Research Progress**

June 2020 - September 2020: Further complete literature review and data retrieval, fully complete the test preparation, complete the test plan, SOP system, CRF table and other data; Application ethics, registration; Coordinate image detection transactions

October 2020 - December 2020: Complete the pre-test and improve the test conditions

January 2021-March 2022: Conduct clinical trials and complete formal clinical trials

April 2022 -- June 2022: Collated data, wrote paper, summarized materials and concluded the thesis.

**XIV. Study participants**

| **Name** | **Title/Professional** | **Task** | **GCP training certificate** |
| --- | --- | --- | --- |
| Li Lingxin | Associate chief physician | Being responsible for the Subject | WZ012020009899 |
| Wang Tingting | Attending physician | Recruitment | / |
| Zhang Yonggang | Therapist | rTMS intervention | / |
| Yang Xirui | Therapist | Evaluation | / |

**XV. References**

[1] Wang Longde. Chinese stroke Prevention and treatment report [R]. Beijing: China Union Medical College Press, 2016.

[2] Sun JH, Tan L, Yu JT. Post-stroke cognitive impairment: epidemiology, mechanisms and management[J]. Ann Transl Med, 2014, 2: 80.

[3] Qu Yanji, Zhuo Lin, Zhan Siyan. A systematic evaluation of the epidemiological characteristics of cognitive impairment after stroke in China [J]. Chinese journal of geriatric cardio-cerebrovascular disease,2013,15(12):1294-1301.

[4] Qu Y, Zhuo L, Li N, et al. Prevalence of poststroke cognitive impairment in China: a community based, cross-sectional study[J]. PLoS ONE, 2015, 10: e0122864.

[5] Renjen PN, Gauba C, Chaudhari D. Cognitive impairment after stroke[J]. Cureus, 2015, 7: e335.

[6] Pasi M, Poggesi A, Salvadori E, et al. Post-stroke dementia and cognitive impairment[J]. Front Neurol Neurosci,2012,30:65-69.

[7] Danovska M, Stamenov B, Alexandrova M, et al. Post-stroke cognitive impairment phenomenology and prognostic factors[J]. Journal of IMAB, 2012, 18: 290-297.

[8] Chinese Guidelines for the diagnosis and treatment of dementia and cognitive impairment. 2018 Chinese Guidelines for the diagnosis and treatment of dementia and cognitive impairment (V) : the diagnosis and treatment of mild cognitive impairment. Chinese medical journal, 2018,98 (17):1294-1301.

[9] Jingjing Xu, Zhongyao Cao, Qinghua Zhang. Effects of repetitive transcranial magnetic stimulation combined with cognitive training on vascular cognitive impairment without dementia [J]. Journal of modern integrated Chinese and western medicine,2018,27(34):3768-3771.

[10] Sebastianelli L, Versace V, Martignago S, Brigo F, Trinka E, Saltuari L, Nardone R. Low-frequency rTMS of the unaffected hemisphere in stroke patients: A systematic review.Acta Neurol Scand. 2017 Dec;136(6):585-605.

[11] Guse B, Falkai P, Wobrock T. Cognitive effects of high-frequency repetitive transcranial magnetic stimulation: a systematic review. J Neural Transm. 2010;117:105-122.

[12] Ma Q, Geng Y, Wang HL, Han B, Wang YY, Li XL, Wang L, Wang MW. High Frequency Repetitive Transcranial Magnetic Stimulation Alleviates Cognitive Impairment and Modulates Hippocampal Synaptic Structural Plasticity in Aged Mice. Front Aging Neurosci. 2019;11:235. doi: 10.3389/fnagi.2019.00235. eCollection 2019.

[13] Lage C, Wiles K, Shergill SS, Tracy DK. A systematic review of the effects of low-frequency repetitive transcranial magnetic stimulation on cognition.J Neural Transm (Vienna). 2016;123(12):1479-1490.

[14] Pobric, G., E. Jefferies and M.A. Ralph, Anterior temporal lobes mediate semantic representation: mimicking semantic dementia by using rTMS in normal participants. Proc Natl Acad Sci U S A, 2007. 104(50): 20137-20141.

[15] Gough PM, Nobre AC, Devlin JT. Dissociating linguistic processes in the left inferior frontal cortex with transcranial magnetic stimulation. J Neurosci. 2005;25:8010-8016.
