# Supplementary material for: The optimum parameters and neuroimaging mechanism of repetitive transcranial magnetic stimulation to post-stroke cognitive impairment, a protocol of an orthogonally-designed randomized controlled trial
Source: PLoS One. 2022 Jul 21;17(7):e0271283. doi: 10.1371/journal.pone.0271283 (PMC9302729; doi:10.1371/journal.pone.0271283)
Supplement: S6 File — (PDF) [file pone.0271283.s006.pdf]

四川大学华西医院专职博士后研发基金2020年度第一批立项项目

序号	科室、中心	姓名	立项编号	项目名称	立项经费（万元）
1	病理研究室	苏征征	2020HXBH001	m^6A修饰对环状RNA circEZH2的表达调控及其在前列腺癌中的作用	15
2	病理研究室	余婷	2020HXBH002	新型叶酸修饰的纳米粒介导MIP-3β免疫基治疗联合化疗的抗乳腺癌作用及机制研究	15
3	超声医学科（超声影像药物研究室）	高彬洋	2020HXBH003	超声介导双靶向双模态显像纳米粒防治动脉粥样硬化易损斑块及合并血栓形成的研究	15
4	超声影像药物研究室	向茜	2020HXBH004	ROS响应的仿生铂基纳米酶声/化动力治疗皮肤黑色素瘤	15
5	放射科	张文静	2020HXBH005	未治疗精神分裂症神经网络异常与外周免疫活动失衡相关性的多模态磁共振研究	15
6	肝脏外科	王海川	2020HXBH006	YAP和TAZ在c-MYC诱导肝癌形成中的功能差异及机制研究	15
7	肝脏外科	谢坤林	2020HXBH007	二硫键异构酶TXNDC12和STAT1相互作用调控肝癌免疫微环境的作用和机制研究	15
8	感染与疫苗研究室	孙美灵	2020HXBH008	青钱柳提取物通过阻断细菌骨架Z环的形成抑制MRSA生长作用的研究	15
9	骨科研究所	陵廷贤	2020HXBH009	掺Cu和rGO的具有微纳杂化结构的BCP支架在感染型骨缺损修复中的应用	15
10	国家成都新药安全性评价中心	王亮	2020HXBH010	线粒体内膜蛋白FAM92A1参与神经突触可塑性及学习记忆的分子机制	15

序号	科室、中心	姓名	立项编号	项目名称	立项经费（万元）
11	国家老年疾病临床医学研究中心	刘晓蕾	2020HXBH011	ω-6/ω-3多不饱和脂肪酸比例在肌少症发病机制中的作用研究	15
12	呼吸与危重症医学科	王德年	2020HXBH012	Intermedin通过调控Src抑制血管内皮炎症的机制研究	15
13	呼吸与危重症医学科	童翔	2020HXBH013	巨噬细胞移动抑制因子（MIF）在肺肌成纤维细胞凋亡抵抗中的作用及调控机制研究	12
14	华西华盛顿线粒体与代谢研究中心	张定坤	2020HXBH014	基于代谢组学技术的多维羧基化碳纳米材料毒性评价研究	15
15	华西-华盛顿线粒体与代谢研究中心	刘月秋	2020HXBH015	地骨皮中α-葡萄糖苷酶抑制活性化合物α-Dimorphecolic acid的胃肠道副作用研究	15
16	华西泌尿外科研究所/华西生物学 医学大数据中心	简钟宇	2020HXBH016	17β-雌二醇—PKA信号通路在肾草酸钙结石形成过程中的机制研究	15
17	华西生物制药研究院	刘强	2020HXBH017	转录调控因子SigS调控金黄色葡萄球菌毒力的机制研究	15
18	康复医学中心	李凌鑫	2020HXBH018	重复经颅磁刺激治疗卒中后认知障碍参数优化及神经影像学研究	15
19	老年医学中心	宋娟	2020HXBH019	基于单细胞转录组测序的人肺泡细胞衰老调控的分子机制研究	15
20	老年医学中心	王艳艳	2020HXBH020	阻塞性睡眠呼吸暂停对腹部外科老年患者围术期神经认知功能的影响及相关机制探讨	15
21	临床磁共振研究中心	徐馨	2020HXBH021	希望对抑郁的保护作用及其脑网络机制研究——基于多模态磁共振成像的研究	15

序号	科室、中心	姓名	立项编号	项目名称	立项经费（万元）
22	麻醉与危重急救研究室	高蕊	2020HXBH022	miRNA-21通过TLR7调控MyD88通路介导固有免疫活化和程序性坏死在术后认知功能障碍中的作用及机制研究	15
23	麻醉与危重急救研究室	周斌	2020HXBH023	视交叉上核中星形胶质细胞的终足样结构调控昼夜节律及相关机制研究	15
24	麻醉转化医学国家地方联合工程研究中心	刘新浩	2020HXBH024	婴幼儿体外循环后肺高压：血小板-血栓素-迷走神经途径介导的机制研究	15
25	美容整形/烧伤外科	袁馨	2020HXBH025	载脂肪干细胞多孔微凝胶促糖尿病创面修复的研究	15
26	泌尿外科	孙光曦	2020HXBH026	Leptin-SGK1-NDRG1信号轴在肾癌-脂肪微环境中介导肿瘤快速演进的机制研究	15
27	泌尿外科/泌尿外科研究所	白云金	2020HXBH027	褪黑素抑制GPX4依耐性铁死亡预防肾草酸钙结石形成的机制研究	15
28	内分泌代谢科	周方励	2020HXBH028	羟基-α-山椒素通过NPC1/NLRP3通路改善NASH的分子机制研究	15
29	皮肤性病科	李仲桃	2020HXBH029	梅勒达病致病基因SLURP1对真皮成纤维细胞的作用与机制研究	15
30	神经内科	彭安娇	2020HXBH030	NRG1-ERBB4信号通路在癫痫及多囊卵巢综合征共病中的作用与机制研究	15
31	神经内科	谭戈	2020HXBH031	基于多模态磁共振的特发性全面性癫痫撤药复发脑网络机制研究	15
32	神经内科	黎彦博	2020HXBH032	TFEB/TFEB依赖性自噬调控NLRP3炎症小体影响缺血性脑损伤的作用机制	15

序号	科室、中心	姓名	立项编号	项目名称	立项经费（万元）
33	神经外科	任艳明	2020HXBH033	自噬调控在颅内非典型畸胎样横纹肌样瘤的作用机制研究	15
34	神经外科	叶曾盼盼	2020HXBH034	氧化应激下Nr4a2介导小胶质细胞M2型极化调控胶质母细胞瘤恶性进展的机制研究	15
35	神经外科研究室	张蜀鑫	2020HXBH035	基于血浆游离DNA表观修饰特征的智能脑肿瘤无创诊断模型研究	15
36	实验肿瘤研究室	赵岗	2020HXBH036	DDX39B通过调控E-cadherin促进非小细胞肺癌生长转移的作用及机制研究	15
37	四川大学生物治疗国家重点实验室	艾潇琳	2020HXBH037	内皮细胞自噬在胶质母细胞瘤肿瘤相关血脑屏障维持中的作用以及机制研究	15
38	胃肠外科中心	方超	2020HXBH038	circWRC通过靶向结合β-catenin调控结直肠癌生长和干性的作用和机理研究	15
39	小儿外科/生物治疗国重	唐勇泉	2020HXBH039	应激性蛋白激酶SGK1通过磷酸化Twist1诱导乳腺癌细胞EMT的机制和应用研究	15
40	心理卫生中心	张霓	2020HXBH040	下丘脑背内侧核促肾上腺质激素释放因子在神经性厌食中的作研究	15
41	心理卫生中心	袁敏兰	2020HXBH041	基于真实世界数据的抑郁症分型以及疗效预测模型构建	15
42	心理卫生中心	李晓晶	2020HXBH042	首发精神分裂症白质纤维异常的神经免疫与炎性机制研究	15
43	心脏大血管外科	陈赛	2020HXBH043	多色荧光图像计数法（MQICM）探究压力负荷下CDK1-AJUBA信号参与心肌成纤维细胞亚型转化的机制	15

序号	科室、中心	姓名	立项编号	项目名称	立项经费（万元）
44	眼科	王力翔	2020HXBH044	恒河猴及食蟹猴玻璃体注射大分子药物所致眼内炎症反应相关因素分析	15
45	移植工程与移植免疫实验室	魏丹凤	2020HXBH045	高亲和性GPA33单链抗体介导的光免疫治疗在结直肠癌中的应用及机制研	15
46	移植工程与移植免疫实验室	王力	2020HXBH046	化疗药物作用下胰腺癌干样细胞中circRNAs的差异性表达与其相关耐药机制的研究	15
47	移植免疫研究室	刘书云	2020HXBH047	肾靶向外泌体递送SOD2治疗AKI-CKD转化的作用及机制研究	15
48	影像医学与核医学	马敏	2020HXBH048	线粒体内膜蛋白在心肌缺血再灌注损伤后心肌焦亡中的相关机制研究	15
49	再生医学研究中心	洪浩	2020HXBH049	组蛋白甲基化酶MLL4调控mTOR自噬通路介导糖尿病心肌病发生的机制研究	15
50	中药药理	李祎铭	2020HXBH050	皮肤前体细胞条件培养基抗光老化作用及机制研究	15
51	肿瘤靶向与免疫治疗研究室	刘小伟	2020HXBH051	新型金纳米笼包载MAPKi增强PD-1抗体在免疫“冷”肿瘤中响应率及机制研究	15
52	肿瘤生物治疗研究室	高兰阳	2020HXBH052	AR新激活剂4OHX靶向FoxA1抗ER^+乳腺癌作用机制研究	15
53	肿瘤生物治疗研究室	肖风来	2020HXBH053	基于任务功能磁共振和多模态影像的青少年肌阵挛癫痫患者学习能力认知脑网络特征研究	15
54	肿瘤生物治疗研究室	蒋静文	2020HXBH054	磷酸丝氨酸转氨酶PSAT1促进肝癌转移的分子机制研究	15

序号	科室、中心	姓名	立项编号	项目名称	立项经费（万元）
55	肿瘤生物治疗研究室	陈婧瑶	2020HXBH055	基于新型子宫内膜癌动物模型筛选鉴定定SETD8抑制剂及其分子机制研究	12
56	肿瘤生物治疗研究室	熊保健	2020HXBH056	镍催化下基于卤化物通过还原偶联制备羧酸酯和酰胺的研究	15
57	肿瘤生物治疗研究室	何林烨	2020HXBH057	IL-28B介导Th17分化在桥本氏甲状腺炎发病中的机制研究	15
58	肿瘤生物治疗研究室	刘莲	2020HXBH058	AHNAK2基因突变在鲜红斑痣发病中的机制研究	15
59	肿瘤生物治疗研究室	聂雯	2020HXBH059	线粒体DNA介导中性粒细胞NOD1/2信号通路激活参与二氧化硅诱导肺部炎性反应的分子机制研究	15
60	肿瘤生物治疗研究室	余佳耘	2020HXBH060	基于乳酸刺激促进CD44表达的肿瘤全细胞疫苗的抗肿瘤机制研究	15
61	肿瘤生物治疗研究室	陈燕	2020HXBH061	STEAP3通过YAP调控铁死亡介导结直肠癌肝脏定植的分子机制研究	15
62	肿瘤生物治疗研究室	黎勇	2020HXBH062	Src/Tubulin双靶点可口服抑制剂KX-01衍生物的设计、合成及其在三阴性乳腺癌中的机制研究及应用	15
63	肿瘤生物治疗研究室	孟婕	2020HXBH063	Tau蛋白调节脑内免疫微环境的机制研究	15
64	肿瘤生物治疗研究室	陈雨文	2020HXBH064	可注射近红外响应性偶氮苯水凝胶用于局部化疗多药耐药乳腺癌	15
65	肿瘤生物治疗研究室	陈飘飘	2020HXBH065	纳米材料和信号放大技术在改善免疫分析性能中的应用研究	15

序号	科室、中心	姓名	立项编号	项目名称	立项经费（万元）
66	肿瘤中心	孙璐	2020HXBH066	NETs相关组蛋白介导肿瘤组织细胞损伤及抑制肿瘤生长的作用机制研究	15
67	转化神经科学中心	徐杨	2020HXBH067	基于JAK-STAT3/NFκB通路SHP2调控PDGFRα磷酸化在星形胶质细胞增殖活化促进骨癌痛中枢敏化中的作用及机制研究	12
