# Supplementary material for: The optimum parameters and neuroimaging mechanism of repetitive transcranial magnetic stimulation to post-stroke cognitive impairment, a protocol of an orthogonally-designed randomized controlled trial
Source: PLoS One. 2022 Jul 21;17(7):e0271283. doi: 10.1371/journal.pone.0271283 (PMC9302729; doi:10.1371/journal.pone.0271283)
Supplement: S7 File — (DOCX) [file pone.0271283.s007.docx]

The first batch of full-time postdoctoral Research and development Fund of West China Hospital of Sichuan University in 2020

| No. | Department | Name | Project number | Project name | Project funds  (Ten thousand RMB) |
| --- | --- | --- | --- | --- | --- |
| 18 | Department of Rehabilitation Medicine | Li Ling-Xin | 2020HXBH018 | Study on parameter optimization and neuroimaging of rTMS treating to PSCI | 15 |
