# Supplementary material for: The optimum parameters and neuroimaging mechanism of repetitive transcranial magnetic stimulation to post-stroke cognitive impairment, a protocol of an orthogonally-designed randomized controlled trial
Source: PLoS One. 2022 Jul 21;17(7):e0271283. doi: 10.1371/journal.pone.0271283 (PMC9302729; doi:10.1371/journal.pone.0271283)
Supplement: Attachment — Submitted filename: Response to Reviewers.docx [file pone.0271283.s008.docx]

Response to Reviewers	PONE-D-21-30462

Dear reviewers:

We have revised the manuscript and replied the 17 items of comments (2 reviewers) point-to-point as below. The detailed change information is showed in the “Main text-Revised Manuscript with Track Changes”.

Thank you for your timely reply!

Your sincerely

Lingxin Li

West China Hospital, Chengdu, China

2022-05-22

Reviewer #1:

Item 1:

Title: please reduce or spell out the use of abbreviation.

Reply:

We have changed and spelled out all of the abbreviations in title. (Line 2 - 12)

Item 2:

Abstract: please include a brief rationale of the protocol; the abbreviation PSCI should be spelled out.

Reply:

We explained the reason and rationale of the protocol and the changes were showed in the objective part of Abstract. The abbreviation PSCI is also be spelled out. (Line 69 – 73; Line 76)

Item 3:

Introduction: please provide a short overview on the pathophysiology and neurobiology of PSCI and, more in general, of vascular cognitive impairment (for a recent comprehensive review, see PMID: 32340195). In particular, the exact relationships between vascular lesion, cognition, and neuroplasticity are not completely understood, although converging findings point out the possibility to identify a panel of markers able to predict cognitive deterioration in the so-called "brain at risk" for vascular or mixed dementia. This will be of pivotal importance when designing trials of disease-modifying drugs or non-pharmacological approaches, such as non-invasive neuromodulatory techniques (including rTMS) in dementia, as very recently reviewed (PMID: 34482205).

Reply:

We added a short overview of the pathophysiology and neurobiology of PSCI in the introduction part and added three references [5-7]. (Line 107 – 115)

Item 4:

Introduction: please state the experimental hypothesis: what did the authors expect? Why and how?

Reply:

We added the hypothesis of the study in the introduction part. (Line 126 – 132)

Item 5:

Methods: did the authors include in the protocol also a sham (fictitious) stimulation procedure?

Reply:

The objective of this study is to probe what the optimal combination of four parameters is, rather than whether the rTMS is valid for the recovery of cognitive function in PSCI patients, so that the sham stimulation control is not designed.

Item 6:

Methods: although a total of 45 patients is acceptable, 5 subjects per group is a rather small sample size.

Reply:

Under the background of the epidemic COVID-19 in the world, the researchers consider that the subject recruitment is difficult. Secondly, although 5 subjects per group is a rather small sample size, the test efficacy is fit with the range and the one-way ANOVA analysis for an orthogonally-designed study. Thirdly, the fMRI data will be analyzed based on three stimulating site subgroups with 15 subjects per group and this met to the recommended sample size medians (14.5 per group) in a literature [Denes Szucs, John Pa Ioannidis. Sample size evolution in neuroimaging research: An evaluation of highly-cited studies (1990-2012) and of latest practices (2017-2018) in high-impact journals. Neuroimage. 2020; 221:117164. PMID: 32679253, [18]]. Hence, the research team decided the sample size as 45 patients in the study. (Line 207 – 214)

Item 7:

Discussion: in the context of the proposed protocol, it should be mentioned that, in some patients plasticity may be considered as an adaptive response to disease progression (thus allowing the preservation of motor programming and execution) but a maladaptive response in others. Please also mention the involvement of metaplasticity in neurological and neuropsychiatric disorders, including stroke and PSCI (PMID: 34276553).

Reply:

It is true that the maladaptive neuroplasticity which might contribute to the dysfunctional remodeling of some specific neural networks in neurological or neuropsychiatric diseases.

We discussed it in the discussion part in our manuscript and added a reference [45]. (Line 498 – 503)

Item 8:

Discussion: pitfalls and limitations of both protocol and technique should be included, together with possible solutions and future research agenda (for some suggestions, please see PMID: 33193753).

Reply:

We evaluated discreetly our study protocol again and discussed the pitfalls and limitations at the end of the discussion part, including the small sample size, the single center study etc. in the end of the Discussion part and added a reference [46]. (Line 504 – 511)

Reviewer #2:

\# Major Issues

Item 9:

Page 10: "active rTMS groups in 1:1 ratio". This ratio is wrong since there are nine treatment groups, not two.

Reply:

This mistake has been corrected as “All participants will be assigned averagely to one of nine paralleled active rTMS groups…” (Line 140 – 141)

Item 10:

Page 10: "This trial will be conducted in line with the consolidated standards of reporting trials (CONSORT) statement...". CONSORT should NOT be used to guide the conduct of trials. These are REPORTING standards. CONSORT does not tell you how to conduct a study. They tell you how to describe it in a consistent fashion. Please change this statement.

Reply:

It has been revised as “This trial protocol will be written in adherence to the standard protocol items: recommendations for interventional trials (SPIRIT) 2013 guidelines [15].”

The reference [12] has also been removed. (Line 143 – 147)

Item 11:

Page 11: "No severe aphasia". How will aphasia be assessed and what is the criteria for severe aphasia used here?

Reply:

No optimal aphasia criteria could evaluate which extent of aphasia is severe aphasia in this trial conducted in China. In practice, we defined the severe aphasia as the global aphasia or in the extent of which will hinder the subjects to complete the MMSE or MoCA assessment pre- or post- treatment. (Line 175 – 176)

Item 12:

Page 12: "informed consent". In this study where patients are cognitively impaired, how will the capacity to understand the study and to give proper informed consent be assessed?

Reply:

The informed consent will be signed by the patient or the authorized person. (Line 178 – 179)

Item 13:

Page 12: The description of the sample size calculations is inappropriate and cannot be confirmed because there is no replicable information provided by the authors. This must be corrected.

Reply:

Because no previous orthogonally designed study provided a significant difference of MoCA or MMSE scores for rTMS treatment to PSCI to estimate the sample size. Hence, we decided to recruit 45 PSCI patients in consideration of the research grant, the recruitment feasibility and the statistic power of fMRI data in neuroimaging research [18]. (Line 207 – 214)

Item 14:

Page 19: The use of one-way ANOVA to analyse paired data (MRI ALFF and FC) is highly inappropriate. This must be changed.

Reply:

For ALFF and FC maps, paired T-test will be used for intra-group comparison and one-way ANOVA analysis will be used for inter-group comparison in the three stimulating site subgroups. The detailed information was shown in the statistical analyses part. (Line 371 – 400; Line 409 – 415)

Item 15:

Page 19: The level of significance must be stated.

Reply:

The multiple comparison correction will be performed using Gaussian random field theory (GRF) with voxel p < 0.005, cluster p < 0.05. The Pearson correlation analyses were used to calculate the correlations between the changes in ALFF and FC with the clinical symptom scores at baseline and the end of the rTMS treatment (p < 0.05). (Line 415 – 427)

Item 16:

*IMPORTANT* There is no assessment of safety. This is a major oversight and needs to be added. This is non-negotiable.

Reply:

We added the adverse events of rTMS treatment as the safety assessment at the end of outcomes part. (Line 307; Line 401 – 405)

Item 17:

*IMPORTANT* Why is there no sham group?

Reply:

The sham group will not be designed in the present study, because the objective of this study is to compare the effect difference in different parameter combination (stimulating site, frequency, intensity and pulse number) of rTMS treating to PSCI and try to find out the optimal parameter combination, but not to confirm whether rTMS is effective for improving the cognitive function in patients with PSCI. Hence, an orthogonally designed study will be conducted.
